# Detection of Human Papillomavirus (HPV) in HPV-Associated Oropharyngeal Squamous Cell Carcinoma: A Review of Diagnostic Approach and Its Importance for the Head and Neck Oncologist

**DOI:** 10.3390/cancers18010056

**Published:** 2025-12-24

**Authors:** Amanda J. Bastien, Daniel Manzoor, Evan S. Walgama, Kevin S. Scher, Julie K. Jang, Justin Moyers, Zachary S. Zumsteg, Allen S. Ho, Jon Mallen-St. Clair

**Affiliations:** 1 Division of Otolaryngology-Head and Neck Surgery, Department of Surgery, Cedars Sinai Medical Center, Los Angeles, CA 90048, USA; amanda.bastien@cshs.org (A.J.B.);; 2 Samuel Oschin Comprehensive Cancer Institute, Cedars Sinai Medical Center, Los Angeles, CA 90048, USA; daniel.manzoor2@cshs.org (D.M.);; 3 Department of Pathology, Cedars-Sinai Medical Center, Los Angeles, CA 90048, USA; 4 Division of Medical Oncology, Department of Medicine, Cedars-Sinai Medical Center, Los Angeles, CA 90048, USA; 5 Department of Radiation Oncology, Cedars-Sinai Medical Center, Los Angeles, CA 90048, USA; 6 The Angeles Clinic and Research Institute, Cedars-Sinai Medical Center, Los Angeles, CA 90048, USA

**Keywords:** histopathology, HPV, IHC, oropharyngeal squamous cell carcinoma

## Abstract

Introduction: Histopathologic assessment of surgical specimens imparts crucial information that is essential for diagnosis, treatment planning and prognostication for patients with Oropharyngeal Squamous Cell Carcinoma (OPSCC). This review explores the range of diagnostic techniques utilized to assess the HPV (Human Papilloma Virus) status in OPSCC. It covers both traditional methods—such as p16 immunohistochemistry, HPV in situ hybridization, and DNA polymerase chain reaction (PCR)—and newer, evolving strategies including circulating HPV tumor DNA analysis and oral HPV DNA/mRNA PCR testing. Discussion: There are currently several histopathologic techniques for the diagnosis of HPV-associated OPSCC. This complexity of care has led to guidelines from numerous authorities (NCCN, ASCO, CAP), which this paper discusses and summarizes for head and neck oncology specialists. Conclusion: The ability to detect HPV in HPV-associated OPSCC is imperative for diagnosis, prognostication, staging, and management of the disease. Advances including liquid biopsy (TTMV-HPV DNA) may be utilized as an adjunct to diagnosis, treatment, and cancer surveillance in the future.

## Simple Summary

The incidence of Oropharyngeal Squamous Cell Carcinoma is increasing in the United States, largely due to the human papillomavirus. Understanding the histopathologic assessment for oropharyngeal squamous cell carcinoma, including the identification of the human papilloma virus (HPV), is essential for successful prognostication and treatment. Several histopathologic techniques are now available for the identification of HPV-associated Oropharyngeal Squamous Cell Carcinoma. These techniques include immunohistochemistry, in situ hybridization and newer approaches such as the liquid biopsy. These variable techniques and approaches have prompted the development of practice guidelines by multiple professional organizations, including the National Comprehensive Cancer Network, the American Society of Clinical Oncology, and the College of American Pathologists. This paper reviews and discusses these guidelines and the current literature surrounding HPV-Associated Oropharyngeal Cancer. Understanding the tumor histopathology is essential for the head and neck oncologist and will better inform multidisciplinary discussions so patients receive maximal patient-centered care.

## 1. Introduction

In 2024, there were approximately 21,000 newly diagnosed cases of oropharyngeal squamous cell carcinoma (OPSCC) in the United States alone [1]. Historically, OPSCC was associated with tobacco and alcohol use. The 1988 Blot et al. study was pivotal in establishing that smoking and alcohol are both independent and multiplicative risk factors for OPSCC [2]. After decades of public health efforts which have included tobacco control, education, and smoking cessation programs, the number of people smoking has significantly decreased from 42.6% in 1965 to 11.6% in 2022 [3]. The data from the Behavioral Risk Factor Surveillance System shows fluctuating reports of heavy alcohol use, with prevalence decreasing from 18.3% in 2011 to 16.0% in 2014, but then having increased to 17.0% in 2017 [4]. Overall, these trends are paralleled by a general decrease in head and neck cancer incidence and mortality [5]. However, the incidence of OPSCC is increasing in the United States. This apparent contradiction supports the notion that OPSCC has a multifaceted etiology [5,6].

The American Society of Clinical Oncology (ASCO) has identified human papillomavirus (HPV) status, smoking history, and TNM cancer staging as significant determinants of overall survival for OPSCC. A patient’s smoking history, especially with several-pack-year history (≥20 pack-years), is associated with worse outcomes regardless of HPV status patients [7,8,9]. There is some evidence reporting an association between marijuana use and OPSCC, but this relationship may be influenced by several other confounding factors. A pooled analysis using the INHANCE consortium demonstrated an elevated risk of OPSCC in patients who smoked marijuana compared to never-users (OR 1.24; 95% CI: 1.06–1.47) [10]. However, after adjusting for potential confounding factors such as HPV exposure, the relationship was weakened (OR, 0.99; 95% CI, 0.71–1.25) [10]. There are also limited studies on the association of periodontal disease and levels of fruit and vegetable intake. Further research is warranted to clarify these associations with the risk of OPSCC [10,11,12].

The current leading risk factor for OPSCC is infection with high-risk subtypes of HPV. HPV is the most prevalent sexually transmitted infection in the United States, with over 50% of the population infected at some point in their lifetime [7,8]. The incidence of HPV-associated OPSCC increased by 225% from 1988 to 2004, while HPV-independent OPSCC declined by 50%. HPV, particularly HPV 16, now accounts for over 80% of OPCSCC diagnoses in the United States [7,8].

HPV is a small, double-stranded DNA virus with over 200 known subtypes. High-risk subtypes include 16, 18, 31, and 33 [13,14]. Other strains identified in oncogenesis include 35, 39, 45, 51, 52, 56, 58, 59, 66, 68, and 73 [15,16,17]. In these HPV-associated malignancies, the proteins E6 and E7 are expressed at high levels. In most patients, the immune system eliminates the HPV infection, but a small number of patients will develop an HPV-related cancer. The time between infection and the development of HPV-associated OPSCC is approximately 10–30 years [17].

The American Joint Committee on Cancer (AJCC) 8th-edition staging system distinguishes between HPV-associated and HPV-independent OPSCC. The AJCC 8th edition employs a relative downstaging for HPV-associated OPSCC to better reflect its more favorable prognosis and unique biology [18]. However, despite this more favorable prognosis, 20% of these patients will recur within 5 years of their initial treatment, thus underscoring the complexity and multifaceted etiology of OPSCC [19,20].

Patient clinical presentations may also vary according to HPV status. Patients with HPV-associated OPSCC often present asymptomatically, but with a neck mass. Evidence supports that HPV-associated OPSCC patients are more likely to present with a neck mass compared to their HPV-negative counterparts. However, patients with HPV-negative OPSCC often have symptoms related to the primary tumor site such as sore throat, odynophagia/dysphagia, and ear pain.

HPV-associated OPSCC tends to present in younger patients (40s to 60s) compared to HPV-negative OPSCC patients who are typically older [21,22,23,24]. Typically HPV-associated OPSCC is four times more likely to be diagnosed in males than females [25]. In the United States, the burden of OPSCC is starting to shift towards older individuals, particularly white men, with projections indicating a substantial increase in cases among those aged 65 years and older [26]. Lower-socioeconomic-status and nonwhite populations have a higher prevalence of HPV-negative OPSCC, whereas higher socioeconomic status is associated with more HPV-associated OPSCC [22]. In the United states the incidence of OPSCC has been shown to be highest in white populations (70.2%), followed by Hispanic (61.3%), Asian (55.8%), and black (46.3%) populations [27]. Its incidence is increasing across all races, but at increased rates in black and Hispanic populations compared to white ones [27].

If there is suspicion of OPSCC, the tissue should be biopsied and sent for histopathologic evaluation. Many times, this can be performed in-clinic transorally or via flexible laryngoscopy. A biopsy under general anesthesia in the operating room does allow for a more thorough examination but may not always be necessary. If the patient is presenting with a neck mass in the setting of nodal metastasis, a biopsy such as a fine needle aspiration should be obtained of the neck node. A small subset of patients may only be diagnosed by needle aspiration of a neck node with no primary tumor site identified; these cases are referred to as “unknown primary.” The NCCN currently recommends HPV testing for all patients diagnosed with OPSCC.

A confirmation of OPSCC should elicit a multidisciplinary treatment team approach. Imaging should be ordered to evaluate the primary tumor and lymph nodes and assess for metastatic cancer. Commonly, a computed tomography (CT) scan is obtained, but an MRI of the neck may assess primary tumor extension and lymph nodes in the neck. These scans should be ordered with intravenous contrast unless the patient has specific contraindications such as allergy or impaired kidney function. Patients should be staged with a CT scan of the chest, but positron emission tomography (PET) is also often used with increased frequency. If the patient is planned for radiation, a dental evaluation should be recommended. Nutrition and speech consultations should be considered as well and have been associated with improved outcomes for head and neck cancer patients [28].

This review explores the range of diagnostic techniques utilized to assess HPV status in OPSCC. It covers traditional methods, such as immunohistochemistry, nucleic acid in situ hybridization, and PCR, as well as newer, evolving strategies including the detection of circulating HPV tumor DNA and oral HPV DNA. Understanding histopathology is essential for all head and neck oncologists, as it directly informs diagnosis, prognosis, and optimal treatment planning in HPV-associated OPSCC. Further understanding of current and upcoming approaches to histopathology will enhance tumor board discussions by enabling head and neck teams to interpret pathologic findings accurately, align them with clinical and radiographic information, and contribute to multidisciplinary treatment decisions for HPV-associated OPSCC.

## 2. Summary of Currently Available Tests for Diagnosis

The College of American Pathologists (CAP) recommends HPV testing on all patients with newly diagnosed OPSCC [24]. CAP recommends the use of p16 immunohistochemical (IHC) staining on tissue from the primary tumor or on a regional lymph node metastasis (when clinically suggestive of an oropharyngeal primary tumor). This recommendation was informed by a 2025 systemic literature review of over 100 studies [29]. The ASCO endorses the CAP recommendation and specifies the use of p16 and the IHC technique as the primary modality to diagnose HPV-associated OPSCC. The NCCN recommends using both p16 IHC and direct HPV testing at clinical centers since concordance testing may vary geographically [30].

Table 1 reviews the current recommendations for HPV testing from multiple authorities.

### 2.1. p16 Immunohistochemistry (IHC)

**Figure 1:**
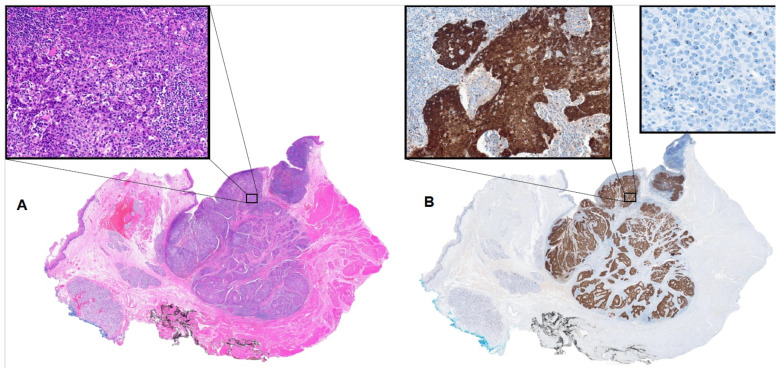
Histologic section of tonsil involved in HPV-associated squamous cell carcinoma. (**A**) Hematoxylin and eosin stain on left with inset showing high magnification of tumor. (**B**) p16 immunohistochemical stain on right with high-magnification inset showing strong, diffuse nuclear and cytoplasmic immunoreactivity (brown stain) in tumor cells, indicative of association with high-risk HPV; high-magnification field from high-risk HPV RNA ISH stain showing reactivity (dark, punctate stain) in tumor cell nuclei, demonstrating direct detection of high-risk HPV RNA.

IHC is an essential diagnostic tool that is readily available and commonly used in most histology laboratories. The technique uses antibodies to detect specific proteins/epitopes expressed in various tissue types. p16 is a protein commonly overexpressed in cells infected with certain HPV subtypes, most notably HPV 16 [24,31,32]. HPV 16 is the most common subtype associated with OPSCC [31,33,34]. p16 IHC is a relatively inexpensive assay that correlates highly with HPV association in OPSCC [24,35,36]. It is currently the principal assay used to diagnose HPV-associated OPSCC. A positive result occurs when more than 70% of tumor cells in a given section demonstrate nuclear and cytoplasmic staining (immunoreactivity) with at least moderate intensity (Figure 1); anything less than 50% is considered a negative result. This requires interpretation by a pathologist, resulting in some interobserver variability [36].

The use of p16 as a surrogate marker for HPV association is not without error; false positives and negatives can occur. Cases of false positivity are due to overexpression of p16 via non-HPV-related pathways [24,37]. In a recent study, 10.9% of p16-positive patients were HPV-negative, and 7.5% of p16-negative patients were in fact HPV-positive [18]. These findings highlight the limitations of relying solely on p16 IHC as a diagnostic surrogate for HPV-positive OPSCC. In the same study, patients with so-called “discordant” results experienced better survival outcomes than patients who tested negative with both p16 IHC and direct HPV testing [18].

While p16 IHC remains a clinically valuable marker, cancer treatment teams must be aware of false positives and false negatives, which can impact prognosis and treatment strategies. Furthermore, the CAP guidelines recommend that after p16 testing, “additional HPV-specific testing may be performed at the discretion of the pathologist and/or treating clinician or in the context of a clinical trial” [24]. In conclusion, this technique is currently one of the most utilized strategies to identify HPV-associated OPSCC, but false positive and negative results do occur. The use of IHC, and thus p16 testing, is widely available in most pathology laboratories and typically remains the first-line testing modality for HPV testing in OPSCC.

### 2.2. HPV-Specific Testing

#### 2.2.1. Nucleic Acid-Based Detection

HPV DNA in situ hybridization (ISH): DNA in situ hybridization (ISH) offers higher specificity (88–100%) but lower sensitivity (83–88%) when compared to p16 IHC [29]. DNA ISH can be useful in assisting for HPV association in discordant cases where p16 is positive but HPV-specific testing is negative. As previously stated, the overexpression of p16 can occur through non-HPV-related pathways. Both ASCO and CAP state that RNA ISH is preferred when available over DNA ISH, as it detects transcriptionally active virus and has a high sensitivity and specificity [24,29,38].

HPV E6/E7 mRNA in situ hybridization (ISH): The use of E6/E7 mRNA RT-PCR is classified currently as the “gold standard” for recognizing HPV-driven tumors by detecting the presence of E6 and E7 mRNA. E6/E7 mRNA RT-PCR has a sensitivity that ranges from 88 to 100%, and specificity ranging from 90 to 100% [35,37,39,40,41]. This method is widely used in research but it is laborious, requires high-quality RNA, and may not be widely available clinically, making it not the first line of testing for HPV compared to IHC [16].

HPV DNA polymerase chain reaction (PCR) is a highly sensitive technique for identifying HPV directly and can be used in conjunction with p16 IHC. This method uses a Taq DNA polymerase and specific primers to amplify target DNA fragments in a sample. The pooled sensitivities for tumor tissue PCR range from 81% to 93% [42]. The use of PCR is more direct and targets specific sequences of HPV before amplification [43,44].

PCR can be used in both fresh and formalin-fixed paraffin-embedded tissue samples, which makes it useful for both retrospective and routine clinical testing. However, DNA PCR alone cannot establish clinically significant (transcriptionally active) infection [24]. This technique allows for both amplification and quantification of specific DNA or RNA sequences. That being said, it is technically demanding and quite costly, making ISH a more commonly used modality.

#### 2.2.2. The Next Frontier: Biomarker-Driven Precision Medicine

The use of the “liquid biopsy” such as circulating tumor tissue-modified viral HPV DNA (TTMV-HPV DNA) and HPV DNA (ctHPV-DNA) is an active area of study. TTMV-HPV DNA has a reported sensitivity of 81% and specificity of 98% [45].

Currently, TTMV-HPV DNA is primarily used for surveillance to detect recurrence of HPV-associated OPSCC, with emerging evidence supporting a potential additive role in diagnosis and monitoring. It is not currently recommended as a replacement for tissue-based HPV testing by authorities [32,38,46]. Patient-reported outcomes support the use of molecular surveillance as a well-accepted technique among patients and may even reduce distress and anxiety in HPV-positive OPSCC patients during their cancer surveillance [47].

Another emerging technique as an adjunct test includes the use of HPV 16 E6 antibodies and oral HPV DNA. HPV 16 E6 is not typically found in the general population but it is present in the majority of patients with HPV 16-associated OPSCC. The reported sensitivities and specificities exceed 90% [33,48,49,50,51]. It has been associated with improved prognosis and may have prognostic value for recurrence similar to the use of TTMV-HPV DNA [34,51,52]. The use of detecting HPV DNA via oral rinse or gargle samples is also undergoing active investigation. This technique is not only less invasive than tissue biopsy but is also convenient for patients. The current sensitivity and specificity have been reported to range from 72% to 92% [53,54]. These techniques have not yet been recommended for population-wide screening due to limitations in disease prevalence as well as the need for further prospective studies [33,53,54]. An overview of these techniques along with their sensitivities and specificities is summarized in Table 2.

Several other areas of study are available for diagnosis, risk stratification, treatment, and monitoring for recurrence. Earland et al. investigated minimal residual disease in lymphatic drainage fluid in HPV-associated OPSCC compared with plasma to assist with risk stratification. Although still undergoing investigation, other tests such as lymphatic drainage may be routinely used in the future alongside histopathology to assist in the stratification of patients who underwent surgery for HPV OPSCC. Other techniques include assessing tumor-infiltrating lymphocytes (TILs), tumor cell multinucleation, and gene expression related to immune response and hypoxia metabolism [66,67]. Using these newer molecular biology tests may assist in the development of a differential diagnosis algorithm, following the model of other neoplasms such as salivary gland carcinoma [68]. The goal of these strategies and techniques is to assist in diagnosis, optimize survival outcomes, and minimize treatment-related morbidity while maximizing quality of life and may allow for personalized therapeutic solutions.

## 3. Discussion

The future landscape of HPV testing will likely go beyond the detection of HPV and instead will yield a more detailed approach as investigators continue to study OPSCC. Currently, p16 IHC and/or HPV-specific testing are typically prerequisites for patient eligibility in numerous ongoing clinical trials, including those investigating treatment de-escalation [69]. They also play a role in the AJCC 8th-edition staging and the downstaging of most HPV-associated cases compared to the 7th edition [46]. As a limitation of the present study, only publicly available and English-language guidelines were included. The future of OPSCC and the study of HPV will assist in stratifying patients, guiding therapy, and integrating molecular biomarkers into clinical decision-making. Research is currently ongoing in refining its application as new biomarkers (such as TTMV-HPV DNA and extranodal extension) are validated.

Many studies have shown extranodal extension (ENE) to be one of the most important pathologic predictors of survival in head and neck cancer [70,71,72]. Its presence suggests overall worse survival as well as increased risk of recurrence and metastasis in HPV-independent head and neck cancers [70,71,72]. In the AJCC 8th edition, ENE was incorporated into the nodal (N) classification for all head and neck cancers except for HPV-associated OPSCC and mucosal melanoma [73]. Recently, Ho et al. demonstrated that metastatic lymph node burden and ENE are the dominant nodal factors driving prognosis in HPV-positive cancers [74]. These findings will likely be incorporated into future staging schemes and assist in treatment discussions for future patients.

The NCCN guidelines currently discuss how a small proportion of cancers in non-oropharyngeal sites, such as the oral cavity and larynx, can be HPV-associated. CAP estimates 2% to 5% of oral cavity squamous cell carcinomas to be HPV-associated. As such, CAP recommends against testing for HPV in these specific tumor sites [29]. The International Agency for Research on Cancer (IARC) approximates that 2% of oral cancers worldwide are caused by HPV [75]. In general, several authorities discourage HPV testing of cancers that occur in primary sites outside of the oropharynx [32,38].

However, the 2025 CAP update reports a conditional recommendation for performing HPV testing on sinonasal SCC. This moderate recommendation is supported by several studies demonstrating that HPV-associated sinonasal SCC comprises approximately 26–30% of all cases. Like HPV-associated OPSCC, HPV-associated sinonasal SCC demonstrates significantly improved prognosis [29]. A National Cancer Database analysis by Amanian et al. reported 5-year overall survival of approximately 62% for HPV-associated versus 35% for HPV-negative sinonasal SCC disease [76]. Unlike OPSCC, CAP guidelines specifically recommend direct testing using RNA ISH while using p16 IHC as a screening tool for confirmation in sinonasal SCC [29,77,78]. The reason for this is a lower HPV prevalence in sinonasal SCC (26% versus >70% in OPSCC). This lower incidence creates a higher discordant testing rate and higher false-positive rate with p16 IHC strategies only. The p16/HPV discordance rates range from 15 to 45% in sinonasal SCC [29,77,78]. The current NCCN guidelines include a statement that HPV testing “may inform etiology” for sinonasal tumors, though the NCCN guidelines do not mandate routine testing for sinonasal SCC at this time [22,30,79].

In summary, HPV testing has become increasingly important in the diagnosis and treatment of specific head and neck subsites, most notably in the OPSCC subsite. HPV-associated SCC represents a distinct biological and separate clinical entity with different prognostic and therapeutic implications. HPV-associated cancers in the head and neck now include OPSCC and sinonasal SCC tumors, but the evidence supporting routine HPV testing in other head and neck subsites remains currently limited and less well established.

Histopathology remains fundamental in the diagnosis and treatment in oncologic care. Having an in-depth understanding of tumor histopathology is essential for head and neck oncologists and will better inform multidisciplinary discussion, guide treatment planning, and optimize patient-centered care and quality of life.

## 4. Conclusions

Routine detection of HPV in OPSCC is essential for accurate diagnosis, prognostication, staging, and optimal multidisciplinary management. There are currently several histopathologic techniques for the identification of HPV-driven disease, and this had led to guidelines from numerous authorities. Currently IHC p16 testing is the first-line testing modality for HPV testing in OPSCC. Emerging tests such as the liquid biopsy (TTMV-HPV DNA) may be utilized as an adjunct to diagnosis, treatment, and cancer surveillance in the future.

## Figures and Tables

**Table 1:** A brief overview of the current recommendations from a variety of authorities on HPV testing in Oropharyngeal Squamous Cell Carcinoma. HPV: human papillomavirus; IHC: immunohistochemistry; ISH: in situ hybridization.

	World Health Organization (WHO) 5th Edition	American Joint Committee on Cancer (AJCC) 8th Edition	National Comprehensive Cancer Network (NCCN) Guidelines	College of American Pathologists (CAP)	American Society of Clinical Oncology (ASCO) Guidelines
HPV testing	No dedicated guideline or position statement on this topic	• p16 testing by IHC or HPV by ISH	• p16 testing by IHC and direct HPV testing	• p16 testing by IHC as primary surrogate marker • In certain cases, proceed with direct HPV testing (typically ISH)	• p16 testing by IHC as primary surrogate marker • In certain cases, proceed with direct HPV testing (typically ISH)

**Table 2:** Overview of select diagnostic techniques utilized to assess HPV status in OPSCC.

Technique	Sensitivity	Specificity	Sources
p16 immunohistochemistry (IHC)	94–100%	79–91%	[45,55]
DNA in situ hybridization (ISH)	85–88%	88–97%	[29,56,57,58]
RNA in situ hybridization (ISH)	88–95%	97–100%	[35,59,60]
HPV DNA PCR	98–100%	84–93%	[42,59,61]
Combining p16 IHC with HPV DNA PCR or RNA ISH	93–97%	96–100%	[35,59,60]
Circulating tumor HPV DNA	81–93%	95–98%	[42,45,62]
Oral HPV wash (oral rinse)	50–84%	85–98%	[53,63,64,65]

## Abbreviations

Oropharyngeal Squamous Cell Carcinoma (OPSCC), HPV (Human Papilloma Virus), polymerase chain reaction (PCR), American Joint Committee on Cancer (AJCC), College of American Pathologists (CAP), immunohistochemical (IHC), American Society of Clinical Oncology (ASCO), National Comprehensive Cancer Network (NCCN), World Health Organization (WHO), in situ hybridization (ISH), circulating tumor tissue-modified viral HPV DNA (TTMV-HPV DNA), HPV DNA (ctHPV-DNA), tumor-infiltrating lymphocytes (TILs), extranodal extension (ENE), and International Agency for Research on Cancer (IARC).
